# Lithium withdrawal and relapse in bipolar disorder when kidney function deteriorates

**DOI:** 10.1192/j.eurpsy.2024.886

**Published:** 2024-08-27

**Authors:** C. De Andrés-Lobo, M. D. C. Vallecillo Adame, T. Jiménez Aparicio, A. Rodríguez Campos, N. Navarro Barriga, M. J. Mateos Sexmero, B. Rodríguez Rodríguez, M. Fernández Lozano, M. Calvo Valcárcel, M. Andreo Vidal, P. Martínez Gimeno, M. P. Pando Fernández, L. Rojas Vázquez, G. Lorenzo Chapatte, M. Ríos Vaquero, A. Monllor Lazarraga

**Affiliations:** Psychiatry, HCUV, Valladolid, Spain

## Abstract

**Introduction:**

Lithium was the first mood stabilizer and today continues to be a first-line treatment in the treatment of bipolar disorder despite its adverse effects, which make it important to monitor blood levels and control kidney function.

**Objectives:**

Presentation of a case of litium withdrawal and relapse in bipolar disorder. Literature review relating to the risk of relapse when lithium treatment is interrupted.

**Methods:**

We present a clinical case of a patient who suffers a deterioration in renal function that requires the withdrawal of lithium and who consequently suffers a relapse. We conducted a bibliographic research of articles in Pubmed on this topic.

**Results:**

A 49-year-old male, with a history of multiple admissions to UHB since the age of 18 with a diagnosis of bipolar disorder and treatment with lithium. Decompensations towards the manic pole have always been related to interruptions in lithium treatment. On several occasions when the patient was feeling well emotionally, he believed himself to be “cured” and abandoned the treatment, triggering a manic episode, showing verbal aggression, increased self-esteem and delusional ideation of harm. Remission was usually achieved with the reintroduction of lithium and the addition of high-dose quetiapine. Between episodes, constant overvalued ideas of economic scarcity seemed to persist, which were accentuated in the form of delusional ideas of ruin in depressive decompensations. After 7 years of stability, control analysis showed blood litemia of 2.2 mEq/L with deterioration of kidney function and generalized tremor was observed, without improvement after serum therapy. He was admitted for dialysis and lithium was suspended. Treatment with valproate was started and a consultation scheduled in a week to adjust the dose. The patient did not attend that consultation and was admitted three days later to Psychiatry Hospitalization showing a challenging attitude, evident dysphoric mood, accelerated speech, with derailments and echolalia. Delusional ideation of harm with auditory hallucinations. Insomnia and hyporexia. Chronic renal failure persisted.

**Conclusions:**

Lithium is a very effective drug but with a narrow therapeutic range that requires adequate monitoring due to the possible consequences of its use at different organs and systems of the body. when lithium is found in the blood at toxic levels with deterioration of kidney function and glomerular filtration fails to recover, lithium treatment should be suspended. Sudden withdrawal of lithium significantly increases the risk of relapse due to rebound effect. More than 50% of patients experience a recurrence within 10 weeks of withdrawal.

**Disclosure of Interest:**

None Declared

